# Insights from agriculture for the management of insecticide resistance in disease vectors

**DOI:** 10.1111/eva.12501

**Published:** 2017-06-29

**Authors:** Eleanore D. Sternberg, Matthew B. Thomas

**Affiliations:** ^1^ Department of Entomology and Center for Infectious Disease Dynamics The Pennsylvania State University University Park PA USA

**Keywords:** global plan for insecticide resistance management in malaria vectors, insecticide resistance, insecticide resistance management, integrated vector management, malaria, vector‐borne diseases

## Abstract

Key to contemporary management of diseases such as malaria, dengue, and filariasis is control of the insect vectors responsible for transmission. Insecticide‐based interventions have contributed to declines in disease burdens in many areas, but this progress could be threatened by the emergence of insecticide resistance in vector populations. Insecticide resistance is likewise a major concern in agriculture, where insect pests can cause substantial yield losses. Here, we explore overlaps between understanding and managing insecticide resistance in agriculture and in public health. We have used the Global Plan for Insecticide Resistance Management in malaria vectors, developed under the auspices of the World Health Organization Global Malaria Program, as a framework for this exploration because it serves as one of the few cohesive documents for managing a global insecticide resistance crisis. Generally, this comparison highlights some fundamental differences between insect control in agriculture and in public health. Moreover, we emphasize that the success of insecticide resistance management strategies is strongly dependent on the biological specifics of each system. We suggest that the biological, operational, and regulatory differences between agriculture and public health limit the wholesale transfer of knowledge and practices from one system to the other. Nonetheless, there are some valuable insights from agriculture that could assist in advancing the existing Global Plan for Insecticide Resistance Management framework.

## 1 INTRODUCTION

Resistance to insecticides is now widespread in the different mosquito species that transmit malaria, dengue, and filariasis, and in other insect species with public health importance (Hemingway & Ranson, 2000; Ranson, Burhani, Lumjuan, & Black IV, 2010; Ranson & Lissenden, 2016). Evidence from laboratory and semi‐field studies suggests that the efficacy of commonly used insecticides, such as pyrethroids, is declining (Macoris et al., 2014; N'Guessan, Corbel, Akogbéto, & Rowland, 2007; Ochomo et al., 2013; Toe et al., 2014; Vontas et al., 2012). Given the importance of insecticides for disease control (Bhatt et al., 2015; Hemingway, 2014; Pluess, Tanser, Lengeler, & Sharp, 2010; White, Conteh, Cibulskis, & Ghani, 2011; World Health Organization, 2006b), it may seem almost inevitable that, unless changes are made, resistance will lead to a resurgence of vector‐borne diseases such as malaria and dengue—although the extent and nature of the epidemiological consequences of resistance remains an open question. In response to this insecticide resistance crisis, the WHO Global Malaria Program has produced a document known as the Global Plan for Insecticide Resistance Management in malaria vectors (GPIRM) (World Health Organization, 2012a), to serve as an action plan for combating insecticide resistance.

Similar to public health, agriculture also has a serious insecticide resistance problem. Insect pests cause chronic and often severe crop loss and, when insecticides fail, there are serious economic losses (Grafius, 1997) and consequences for food security. The evolutionary forces, mechanisms of resistance (for example, mutations in the sodium ion channel gene [Soderlund & Knipple, 2003]), and even the insecticides used are often the same, regardless of whether an insect is an agricultural pest or a vector of human disease. Thus, we might expect there to be common ground and perhaps common solutions for insecticide resistance in agriculture and public health. Here, we explore whether this is indeed the case, and whether insights from agriculture might help in addressing the challenges of insecticide resistance in public health. For our approach, we use the GPIRM as a framework. The GPIRM is structured around five interrelated activities or “pillars” that outline a specific, global strategy for managing insecticide resistance in malaria vectors, namely: (i) plan and implement insecticide resistance management strategies, (ii) ensure proper resistance monitoring, (iii) develop new vector control tools, (iv) fill knowledge gaps, and (v) ensure that key enabling mechanisms (advocacy, human, and financial resources) are in place. We use a comparative approach based around these GPIRM pillars to better characterize the challenges and opportunities for addressing the resistance crisis in public health.

## 2 PILLAR I: INSECTICIDE RESISTANCE MANAGEMENT STRATEGIES

### 2.1 Reducing insecticide use

The greater the use of insecticides, the greater the selection for insecticide resistance and the faster insects become resistant. In agriculture, integrated pest management (IPM) reduces reliance on insecticides by drawing on a much wider range of control measures, including biological control, cultural practices, host plant resistance, semiochemicals, surveillance, and monitoring. Consequently, IPM reduces selection for insecticide resistance and serves as one of the main strategies for resistance management (although it is noteworthy that insecticide resistance is not the only driver for the development and uptake of IPM in agriculture; reducing the use of chemical insecticides for environmental and health concerns was and is an important motivator [Kogan, 1998]).

Integrated vector management (IVM) is the equivalent of IPM, and, like with IPM, a principal tenet is the adaptive, evidence‐based integration of multiple chemical and nonchemical control measures (World Health Organization, 2008, 2012b). The goal of IVM is to make the best use of available resources for vector control, and ultimately for disease control, and it should be viewed as foundational for the management of insecticide resistance in public health (Chanda, Ameneshewa, Bagayoko, Govere, & Macdonald, 2017; Thomas et al., 2012). We would argue that IVM should be widely adopted regardless of current evidence for insecticide resistance in a given area. As we discuss further in later sections, detecting resistance before it becomes a problem is a difficult task; there is much to be gained and little to be lost in implementing IVM regardless of whether there is evidence for resistance in the area.

Implementation of IVM will certainly require buy‐in from various stakeholders (e.g., National Malaria Control Programs, various NGOs), as well as cross‐sectional collaboration and capacity building, and the WHO recognized this as an essential component of IVM (World Health Organization, 2008, 2012b). On a more basic level, however, it will first require strengthening and diversifying methods of vector control that are complimentary to the core insecticide‐based approaches of indoor residual spraying (IRS) and long‐lasting insecticidal nets (LLINs). Alternative approaches, a number of which are already in the later stages of development, include house screening (Kirby et al., 2009), eave tubes (Sternberg et al., 2016), attractive toxic sugar baits (Müller et al., 2010), spatial repellents (Achee et al., 2012), entomopathogenic fungus‐impregnated targets (Heinig, Paaijmans, Hancock, & Thomas, 2015), mass trapping (Homan et al., 2016), and diverse strategies targeting zoophilic vectors (Chaccour et al., 2015; Massebo, Balkew, Gebre‐Michael, & Lindtjørn, 2015; Waite et al., 2017). These new approaches, together with established strategies of larval source management (Tusting et al., 2013), could all potentially contribute within an IVM framework.

### 2.2 Increasing insecticide diversity

Another strategy for slowing insecticide resistance, not mutually exclusive with reduced reliance on insecticides, is varying the use of different insecticides over space and time; for example, mixtures (co‐formulations of two or more insecticides with different modes of action), rotations (alternating between two or more insecticides over time), or mosaics (use of different insecticides in neighboring geographic areas) (see also Huijbin, & Paaijmans, In Preparation). Numerous empirical and theoretical studies in agricultural systems have explored these different strategies (Immaraju, Morse, & Hobza, 1990; MacDonald, Surgeoner, Solomon, & Harris, 1983; Pimentel & Burgess, 1985), reviewed by Tabashnik (1989), and perhaps the only universal conclusion is that the success of a specific strategy is hugely dependent on the specifics of a pest–crop system, including insect genetics, behavior, population dynamics, and the chemical nature of the insecticides and their formulation.

For example, insecticide mixtures rely on redundant killing so that even if an insect is resistant to one of the insecticides used in the mixture, the second insecticide will still kill it. One review of xenobiotics (both antimicrobial drugs and insecticides) found that combination strategies (mixtures) were as good as or better than alternative strategies in many cases (REX Consortium, 2013). However, there are a number of biological and operational caveats. Co‐formulations can be difficult to develop and mixtures only offer an advantage as long as the component insecticides both continue to work, such that the efficacy of mixtures for controlling resistance depends on the co‐persistence of the component insecticides. Additionally, resistance alleles in the insects must be rare and fully recessive, to avoid strong selection for double heterozygous individuals and the rapid evolution of resistance to both insecticides (Curtis, 1985; Tabashnik, 1989). Similar caveats exist for the use of mosaic and rotation strategies, and modeling efforts aimed at evaluating different strategies for insecticide resistance management have demonstrated that the outcomes are highly sensitive to parameters that are system specific (Lenormand & Raymond, 1998; Slater, Stratonovitch, Elias, Semenov, & Denholm, 2016), such that there is no universal best strategy. Consequently, insecticide resistance management plans are generally specific to a single pest species and crop (e.g., Colorado potato beetles on potato crops in the United States [Huseth et al., 2014], or pollen beetles on oilseed rape in Europe [Slater et al., 2011]).

Because of the situation‐specific nature of insecticide resistance management plans, public health may have an advantage over agriculture. Agricultural pest management is concerned with hundreds of species, and in many cases, detailed data are not available for each pest species (and potential nontarget organisms). Compared to agriculture, public health is concerned with a small number of insect species. This should enable a strongly data‐driven approach to selecting resistance management strategies, but in reality, very little is currently known.

GPIRM does highlight the success of a rotation‐based management strategy in one public health example: the West African Onchocerciasis Control Program (OCP). Onchocerciasis or “river blindness” is a helminthic infection transmitted by black flies. Not surprisingly, given the evidence from agricultural systems, the success of the program can be attributed in part to a consideration of the specific ecology of the system when designing the rotation scheme (Curtis, Hill, & Kasim, 1993). Importantly, the OCP had access to six larvicides covering three chemical classes (three organophosphates, a pyrethroid, and a carbamate), plus a biological (*Bacillus thuringiensis*, or Bt), all of which could be rotated with relative ease, as the insecticide treatment targeted fast‐moving water (the preferred habitat of black fly larvae) where the insecticides were washed out before the next treatment. In contrast to the OCP, malaria vector control relies heavily on LLINs, which are almost exclusively treated with pyrethroid insecticides. In 2015, 53% of people at risk for malaria worldwide had access to an LLIN. Only 3.1% of people at risk received protection from IRS, where a nonpyrethroid can be used. Although it is recommended that a nonpyrethroid is used for IRS in areas where it is used in combination with LLINs (World Health Organization, 2012a, 2014), pyrethroids are still used for the majority of IRS (World Health Organization, 2016b). In the vast majority of locations, therefore, strategies based on multiple insecticides are effectively impossible at present (although the NGenIRS partnership (http://www.ngenirs.org/), led by the Innovative Vector Control Consortium, is one example of ongoing work to address this problem).

### 2.3 Summary

- Insecticide resistance management (IRM) strategies are urgently needed for vector control.
- The feasibility of IRM depends on having multiple insecticides and vector control tools; vector control is currently dependent almost exclusively on a single insecticide class.
- The success of an IRM strategy is highly dependent on ecological and biological specifics; there is not yet a solid evidence base of recommending one strategy over another.
- If control is achieved via the use of multiple tools with diverse modes of action, not only will selection for insecticide resistance be reduced but also, the impact of resistance will likely be less severe. The pending resistance crisis creates an urgent need to develop and implement integrated, multitactic IVM strategies that parallel IPM in agriculture.

## 3 PILLAR II: RESISTANCE MONITORING

### 3.1 Selecting the appropriate targets for monitoring

Currently, monitoring of insecticide resistance in vectors is perhaps most limited by a lack of clarity on what to measure, and how. Molecular markers are an attractive option for monitoring resistance because they hold the possibility of more rapid throughput and greater resolution of the resistance landscape (Donnelly, Isaacs, & Weetman, 2016). Insects can be saved for later analysis, and molecular testing is easier to standardize than phenotypic bioassays. However, the link between resistance genotype and phenotype can be complex and is often poorly characterized. Knockdown resistance (*kdr*) is perhaps one of the best‐characterized target site mutations in both agricultural pests and disease vectors, and yet, there is still no unambiguous evidence that *kdr* frequencies are predictive of operationally significant levels of pyrethroid resistance in vectors (Hemingway, 2014). For example, pyrethroid‐based IRS was still used effectively for malaria control on Bioko Island despite high levels of *kdr* in the *Anopheles gambiae* population (Hemingway et al., 2013). Additionally, in some cases, *kdr* is essentially fixed and therefore has very little explanatory power.

Phenotypic bioassays have already informed a number of successful resistance management plans in agriculture (Alyokhin et al., 2015; Denholm, Cahill, Dennehy, & Horowitz, 1998), but bioassays come with their own set of challenges. Results of supposedly highly standardized WHO bioassays have been shown to fluctuate significantly between multiple tests performed on the same population (Badolo et al., 2012), which makes it particularly difficult to detect relationships between bioassay results and other variables. Until relatively recently, the focus of bioassays was to monitor the frequency of resistance phenotypes, which failed to distinguish, for example, between moderate (two‐ to fivefold) resistance and the 100‐ to 1000‐fold resistance to pyrethroids that has been reported in *Anopheles* mosquitoes in parts of West Africa (Hemingway, 2014). The need to monitor intensity of resistance has consequently begun to receive more attention (Bagi et al., 2015), which may provide a more nuanced understanding of resistance in disease vectors. Importantly, however, none of the established bioassay methods for measuring resistance have associated significantly with epidemiological endpoints such as prevalence of malaria (Wondji et al., 2012; World Health Organization, 2016a). There is one crucial difference between agriculture and public health, namely that the former uses insecticides to control insect pest populations while the latter uses insecticides to control disease transmission by insects. However, in both cases, it is important to note that bioassay results serve as a warning sign, and not as a guarantee of either uncontrolled crop damage or disease resurgence.

### 3.2 Resistance monitoring for resistance management

Even with an operationally significant signal of resistance, there is another practical limitation on resistance monitoring. Insecticide resistance alleles are, in theory, initially rare. Monitoring must be extremely sensitive and changes must be made while resistance allele frequencies are still low—a practical limit proposed in the agricultural literature suggests that monitoring must be able to detect resistant individuals at frequencies of 1% or lower (Roush & Miller, 1986). Because it is so difficult to achieve adequate sensitivity, in many cases by the time resistance is detected, it will already be too late to implement resistance management measures and slow the spread of resistance.

However, difficulties with monitoring resistance need not prevent the implementation of IRM strategies and it is possible, and perhaps even preferable, to implement strategies that do not rely on early detection of resistance. Although the OCP did conduct frequent entomological surveys on black flies, the program relied on set rotation program determined beforehand based on cost as well as other factors (Hougard et al., 1993). In other words, the decision on which insecticide to use was not based on the detection of resistance, other than the initial observation that control with a single insecticide was failing due to resistance (Curtis et al., 1993). Likewise, the successful management of resistance in white flies in Israel and the United States depended on a set rotation program that reduced the number of insecticide treatments per year and limited the use of multiple insecticides with similar chemistries (Denholm et al., 1998; Palumbo, Horowitz, & Prabhaker, 2001)—although the use of certain insecticides was modified or suspended over time in response to resistance. In general, changing insecticides in response to resistance (i.e., responsive alternation) tends to perform more poorly than preset rotations (REX Consortium, 2013). The best strategy may be to use resistance monitoring to inform *which* insecticides can be used in a rotation, but not *when* they should be used.

### 3.3 Summary

- There is a need for more effective resistance monitoring tools that enable the functional (epidemiological) impact of resistance to be determined.
- Effective resistance monitoring tools should, in principle, aid the development and evaluation of IRM strategies.
- Resistance monitoring has historically been more useful for documenting failures than for avoiding them.
- Imperfect resistance monitoring tools are not prohibitive to successful resistance management.

## 4 PILLAR III: NEW VECTOR CONTROL TOOLS

### 4.1 New insecticides

The availability of new insecticides is far more limiting in public health than in agriculture. The agricultural chemical sector is an US$ 8 billion per year industry (Hemingway, 2014) that often produces novel insecticides with more than one application—that is, that can be used on more than one type of crop or pest. Large markets encourage innovation and help maintain a robust insecticide pipeline. In contrast, the market size for vector control products is only US$ 0.2–0.7 billion/year (Hemingway, 2014) and as a relatively small market, it is difficult to meet the need for a rapid return to cover large, up‐front research and development costs. The Innovative Vector Control Consortium (IVCC) is a public–private product development partnership with the remit of alleviating the economic pressures that work against the development of new vector control insecticides (Hemingway, Beaty, Rowland, Scott, & Sharp, 2006). Since its launch in 2005, the IVCC partnership has produced two new IRS formulations, including one with an active ingredient belonging to a chemical class not previously used in public health (Actellic CS). The partnership is also in the final stage of development for a number of actives that would diversify the chemical classes available to public health (Hemingway et al., 2016; Innovative Vector Control Consortium (IVCC), n.d.; Oxborough et al., 2014; Rowland et al., 2013). Despite the contribution of IVCC, it is unlikely that public health insecticide availability and diversity will ever match that of agriculture.

In fact, the public health insecticide product pipeline is not separate from the agricultural product pipeline. At this point, the active ingredients used in vector control are essentially repurposed agricultural insecticides. Many of these insecticides, or insecticides with the same modes of action, continue to be used on crops, and there is evidence that agriculture is an important source of selection for insecticide resistance in disease vectors (Akogbéto, Djouaka, & Kindé‐Gazard, 2006; Chouaïbou et al., 2016; Curtis, Miller, Hodjati, Kolaczinski, & Kasumba, 1998; Nkya, Akhouayri, et al., 2014; Nkya, Poupardin, et al., 2014).

An observational study in Tanzania found that in agricultural areas, the use of pesticides from various chemical families appeared to select for metabolic and cuticular resistance mechanisms against a wide range of insecticides in malaria mosquitoes, including resistance to pyrethroids and carbamates used for vector control. In urban areas, the use of LLINs specifically appeared to be selecting *kdr* mutations, but with environmental pollutants potentially favoring the selection of some detoxification enzymes (Nkya, Akhouayri, et al., 2014). An experimental evolution approach found that mosquito larvae recurrently exposed to an agricultural pesticide mixture could develop adult resistance mechanisms against vector control insecticides. Transcriptomics revealed that a broad range of biological functions was affected, including detoxification, cuticle, gene regulation, and nervous system function (Nkya, Poupardin, et al., 2014). These data may help explain why resistance to insecticides is developing so rapidly in malaria mosquitoes in Africa. Recent modeling work demonstrated that, as exposure to insecticides in the environment increases (for example, due to agricultural use), increasing the number of insecticide‐treated bed nets in an area has less of an impact on the time that it takes for vector populations to become resistant (Birget & Koella, 2015). This suggests that, under certain conditions, modifying public health use of insecticides might not be sufficient to slow or manage resistance evolution in disease vectors.

The insecticides repurposed from agriculture for vector control are, for the most part, fast‐acting lethal insecticides. As a consequence, rapid mortality has become an accepted part of the target product profile (TPP) for public health insecticides. However, the ultimate endpoint of vector control is to prevent disease transmission and this does not necessarily require rapid mortality. Recent empirical and theoretical work demonstrates that insecticides that work more slowly, so‐called late‐life‐acting (LLA) insecticides, can still reduce transmission (Koella, Lynch, Thomas, & Read, 2009; Read, Lynch, & Thomas, 2009; Viana, Hughes, Matthiopoulos, Ranson, & Ferguson, 2016). Because it takes 10–14 days for malaria parasites to develop within a mosquito, killing older mosquitoes can reduce transmission even if the density of young mosquitoes remains unchanged. In other words, unlike in agriculture, the desired endpoint of disease control can be achieved by changing age structure and not necessarily population density. Importantly, killing mosquitoes later in life has less of a fitness cost than killing mosquitoes early in life and hence, it has been proposed that LLA insecticides should impose less selection for resistance than fast‐acting insecticides. Recently proposed “evolved spatial repellents” (ESR) would be another potential strategy where the aim is disease control and not insect control. In this strategy, a partially effective repellent is paired with a highly toxic insecticide. By deflecting a proportion of vectors prior to contact with the insecticide, this method could select for aversion to the repellent while delaying the evolution of resistance to the toxic insecticide (Lynch & Boots, 2016). In some ways, this is conceptually similar to the “high‐dose/refuge” strategy from agriculture (discussed in more detail under Pillar V), where a pool of susceptible insects is intentionally maintained to preserve susceptible alleles in the population.

In agriculture, decisions to treat with insecticides are usually based on assessments of pest density in relation to predetermined Economic Threshold Levels (ETLs), which define the point at which return on investment in control outweighs the expected costs of pest damage (Higley & Pedigo, 1996). If a pest is at the ETL, remedial action is required and generally achieved with fast‐acting contact insecticides. However, implicit in the concept of an ETL is the “management” of populations and not just “control.” Certain systemic insecticides (i.e., insecticides present in plant tissues), biological insecticides, and some pheromone‐ or hormone‐based products do not induce rapid mortality but work in a more preventative fashion. Together with a suite of other IPM tactics, these products can be used to keep populations below the ETL. Whether it is possible to operationalize alternatives to fast‐acting insecticides for vector control remains to be fully tested, but broadening the TPP and creating new TPPs that consider alternative strategies could encourage a greater diversity of products in the public health portfolio.

Another key feature of a TPP is product persistence. In vector control, there is a strong preference for products to last as long as possible so that retreatment frequency, and cost, can be reduced. The current target for LLINs is 3 years and 20 washes; for indoor residual spraying (IRS), the aim is 6 months to a year (World Health Organization, 2006a, 2013). There are relatively few examples in agriculture where the targets for persistence are so extreme. In part, this is because retreatment tends to be less cost‐prohibitive. Additionally, concerns over health and environmental impacts favor less persistent products for agricultural use. Evidence from agricultural systems also suggests that prolonged decay can facilitate the spread of resistance alleles (Roush, 1989; Roush & McKenzie, 1987). Perhaps the most effective persistence profile from a resistance management perspective is a sustained high dose with rapid decay—in other words, “hit them hard or not at all” (REX Consortium, 2013). Efforts to develop long‐lasting products for vector control might be counterproductive with regard to resistance management if the result is a long and shallow decay curve. The ideal persistence profile from a resistance management perspective needs to be balanced, however, with the need to provide continuous protection from disease. A product with rapid decay could leave people unprotected from disease if too much time elapses between treatments.

### 4.2 New delivery methods

The emphasis on long‐lasting products in public health might also make it more difficult to implement insecticide resistance management strategies, such as rotations. Once LLINs have been distributed, for example, it is untenable to remove them and switch to a different insecticide. The unintentional exposure of populations to multiple insecticides due to poorly coordinated rotation has been associated with rapid development of insecticide resistance in agricultural systems (e.g., Zhao et al., 2006). If LLINs are indeed selecting for insecticide resistance in vectors, and not agricultural use of insecticides as discussed in the previous subsection, then the longevity of LLINs could be counterproductive to resistance management efforts. Although there is currently little empirical evidence to support one insecticide use strategy over another (e.g., rotation vs. mosaics) for vector control, it is likely that IRM will require new, more flexible delivery methods for insecticides, in addition to new active ingredients and non‐insecticide‐based control methods.

### 4.3 Summary

- The new product pipeline for vector control is limited by a lack of economic incentives; substantial changes either to the supply side (e.g., through product development partnerships like IVCC) or the demand side are necessary to diversify options.
- Current TPPs that favor fast kill and persistence are, at best, limiting the products available for insecticide resistance management and, at worst, creating conditions that maximize selection for resistance.
- Defining outcome‐based TPPs, where the outcomes are reduced disease transmission while still slowing insecticide resistance, could encourage development of a broader range of products.

## 5 PILLAR IV: FILLING KNOWLEDGE GAPS

One of the aims of the GPIRM was to guide the research priorities for vector control. The development of new tools (Pillar III) is part of that research agenda, but GPIRM also covers knowledge gaps in resistance mechanisms, the operational significance of insecticide resistance, and the impact of IRM strategies.

### 5.1 Epidemiological consequences of resistance

Despite the spread of phenotypic (based on bioassays such as the WHO tube test) and genotypic (based on genetic markers such as *kdr*) resistance, the evidence for control failure due to resistance is equivocal, although perhaps clearer for IRS than for LLINs (Bradley et al., 2017; Maharaj, Mthembu, & Sharp, 2005; Wondji et al., 2012; World Health Organization, 2016a; see also Huijbin, & Paaijmans, In Preparation).

Part of the difficulty is that there is a complex relationship between resistance and epidemiological outcomes (Thomas & Read, 2016). In agriculture, resistance leads to the growth of pest populations, which leads to more pest damage to crops. In public health, population growth does not necessarily translate to more disease transmission. For example, increasing densities of vector populations might influence phenotypic traits that make better or worse disease vectors (Moller‐Jacobs, Murdock, & Thomas, 2014; Russell et al., 2011; Shapiro, Murdock, Jacobs, Thomas, & Thomas, 2016). Additionally, there is evidence of subtle effects of insecticide exposure that might impact mosquito population age structure, rather than density (Viana et al., 2016). There is also evidence for interactions between insecticide resistance and parasite infection and development, which could impact mosquito transmission potential irrespective of density (Alout et al., 2014; Rivero, Vézilier, Weill, Read, & Gandon, 2010).

### 5.2 Evaluating resistance management strategies

Another difficulty, as discussed in the GPIRM, is that randomized controlled trials (RCTs) are the gold standard for demonstrating the epidemiological impact of an intervention, but it is not possible to randomly allocate resistance levels among treatments. It is possible to randomly allocate resistance management strategies, but such trials are costly, logistically difficult, and extremely time and labor intensive. To our knowledge, there is only one field trial aimed specifically at testing resistance management strategies against malaria mosquitoes (Penilla et al., 2007). Perhaps worryingly, this trial revealed no clear benefit of either insecticide rotations or mosaics in slowing the spread of resistance when compared to monotherapy. However, these results do not necessarily mean that rotations or mosaics cannot be effective, but rather highlights a critical gap in understanding when, how, and why such strategies might work.

### 5.3 Summary

- The epidemiological impacts of insecticide resistance can be complex and at present, there is mixed evidence that control is being substantially compromised due to resistance.
- Vector density is not the only factor that determines malaria epidemiology; additional factors may obscure the relationship between insecticide resistance and malaria.
- Field testing of IRM strategies is difficult but necessary. GPIRM makes numerous references to approaches such as rotations, mixtures, or mosaics, as if they are proven strategies and have equal merit. At present, there are no sound empirical data to inform the selection of any one strategy over another.

## 6 PILLAR V: ENABLING MECHANISMS

### 6.1 Long‐term value over short‐term cost

Arguably, one of the most successful resistance management program in agriculture is the management of Bt resistance in genetically modified (GM) crop in the United States (Bates, Zhao, Roush, & Shelton, 2005; Tabashnik, Gassmann, Crowder, & Carriére, 2008; Tabashnik et al., 2003). This is a high‐dose/refuge strategy based on expression of high doses of Bt toxin within GM crops, combined with non‐Bt refuges, which can be non‐GM cotton or corn or even another plant used by the target pest. The high dose of Bt is designed to kill fully susceptible and heterozygote‐resistant insects. Any homozygote‐resistant individuals will be initially rare, and there is a high probability that they will mate with the numerically dominant susceptible individuals emerging from the non‐Bt refuge. These matings result in heterozygotes that are functionally susceptible to the Bt crop, effectively clearing resistance alleles from the pest population. Factors that have likely contributed to the success of this strategy include low initial frequencies of resistance alleles, recessive inheritance of those resistance alleles, and fitness costs associated with resistance (Tabashnik et al., 2003). Again, we see that the success of a management strategy is dependent on the biology of the system.

The recommended refuge size is variable, but a farmer might need to dedicate nearly half of his or her cropping area to plants that generate the very pest that he or she is trying to control (Bates et al., 2005; Bourguet, Desquilbet, & Lemarié, 2005; Dove, 1999). This requires the farmer to place greater value on the long‐term gain of preserving Bt as a pest management tool over the short‐term financial loss from planting a refuge of non‐Bt plants. While some degree of crop loss is acceptable in agriculture, the only acceptable human disease burden in zero. As discussed in the previous section, however, disease control does not necessarily require complete eradication of vectors. Products or control strategies that maintain a pool of susceptible and nontransmitting vectors would be a useful addition to the vector control toolbox.

### 6.2 Susceptibility as a public good

Because most pest insects are mobile, and resistance it not limited to one field or farm (Slater et al., 2016), IRM strategies require buy‐in from the entire community using an insecticide. The high dose/refuges have both a relatively large cost to implement and will likely have a negligible impact if only a few members of the community implement them. This creates a tragedy of the commons: the pool of susceptible insects is the common good and farmers must prioritize the long‐term gain from preserving the pool of susceptible insects over the short‐term, personal gain of planting only Bt crop. The cost to the farmers from planting a refuge does make compliance with the management strategy difficult to achieve without any external pressures. Government oversight is one approach to preserving a public good; in the United States, the Environmental Protection Agency (EPA) mandates the use of refuges with all Bt crops, including size and placement of the refuges, but ensuring compliance in the end users (i.e., farmers) falls to the companies that make and sell the products (Bates et al., 2005; Dove, 1999). Methods for encouraging compliance include education on the benefits of resistance management strategies, but it also includes monitoring and temporary blocks against noncompliant farmers to prevent them from buying Bt seeds. Extensive outreach and government oversight were also essential in another management strategy using insecticide rotations to control white flies in Israel. In addition to educational programs and outreach, as with Bt, the Israeli government also centralized the sale of insecticides with the Israeli Cotton Board (Denholm et al., 1998). Although government regulation has been an approach used successfully for enforcing good management practice in several agricultural examples, it is worth noting that there can be nongovernmental approaches to preserving a public good like susceptibility, such as community enforcement of sustainable use practices. However, given that LLIN and IRS procurement and distribution are typically decided on a national level or higher, there could be an even greater opportunity than in agriculture for governmental oversight to preserve susceptibility as public good.

### 6.3 Summary

- Vector control is currently dominated by short‐term economics that emphasizes product “cost” rather than “value,” and favors minimum cost for maximum coverage.
- Because insects are mobile, IRM requires buy‐in from most or all users of an insecticide.
- There are also no incentives or regulatory framework to support the concept of susceptibility as a public good in vector control; susceptibility to insecticides should be considered a public good and protected accordingly.

## 7 CONCLUSIONS

Agriculture has been combatting resistance evolution for decades. There are some significant success stories but these stand out, in part, because they are relatively rare. It is important to recognize that agriculture does not have all the answers; even today, insecticide resistance is still a serious problem for controlling agricultural pest species.

There are also fundamental differences between insect control in agriculture and public health. Appreciating these differences helps identify both opportunities and limits for knowledge transfer. For example, agriculture has a much larger insecticide discovery pipeline than public health. This pipeline enables agriculture to deal with the consequences of resistance by moving from product to product—that is, practicing resistance mitigation rather than resistance management. This strategy works, as long as the pipeline is sufficiently open‐ended and new products satisfy regulatory and economic constraints. With a robust pipeline, resistance in the target insect population can even serve to remove older products from the market and replace them with new products. This replacement benefits companies if a new product that is still covered by intellectual property protection replaces a generic product. If regulators have tightened constraints over time, then the new product may also be safer or have a more desirable profile than the older product, which benefits the public. Vector control does not have an equivalent pipeline to agriculture, or an equivalent diversity of products, and it likely never will. New insecticides can initially control resistant vectors (N'Guessan et al., 2014; Ngufor et al., 2014), but if they are used in the same way as existing insecticides (i.e., for mitigation rather than management), resistance will inevitably evolve. Because there are fewer opportunities for replacement of products, IRM is perhaps even a greater priority for public health than for agriculture, with integrated vector management as the foundation of IRM.

Another important difference is that the goal for agriculture is to the keep pest population densities below a set ETL, while the goal for public health is to prevent disease transmission. This means that reducing insect population density is important for public health only in so far as it reduces the number of infectious vector bites. Alternative methods that, for example, change population age structure rather than density can and should be considered for vector control.

Resistance management in agriculture has been most successful in situations where susceptibility has been recognized and regulated as a public good. The current funding model for vector control emphasizes the direct cost of tools or strategies. For IRM strategies to succeed in public health, there needs to be a shift away from a “cost‐based” model (i.e., choosing vector control tools or strategies based on direct cost) and toward a “value‐based” model (i.e., factoring in the benefit of preserving susceptibility). Such changes will require the development of appropriate regulatory frameworks. Although these are not easy changes to affect, they are essential to support the future development and implementation of IRM in public health.

## DATA ARCHIVING STATEMENT

There are no data associated with this manuscript to archive.
